# Low-Grade Inflammation and Increased Arterial Stiffness in Chinese Youth and Adolescents with Newly-Diagnosed Type 2 Diabetes Mellitus

**DOI:** 10.4274/jcrpe.2187

**Published:** 2015-12-03

**Authors:** Xin Li, You Ping Deng, Miao Yang, Yu-Wen Wu, Su-Xin Sun, Jia-Zhong Sun

**Affiliations:** 1 Wuhan University Faculty of Medicine, Zhongnan Hospital, Clinic of Endocrinology, Wuhan, China; 2 Wuhan University Faculty of Medicine, Zhongnan Hospital, Clinic of Pediatrics, Wuhan, China

**Keywords:** Low-grade inflammation, youth and adolescent, type 2 diabetes mellitus, Arterial stiffness

## Abstract

**Objective:**

To investigate the relationship between low-grade inflammation (LI) and increased arterial stiffness in Chinese youth and adolescents with newly-diagnosed type 2 diabetes mellitus (T2DM).

**Methods:**

Ninety-eight subjects aged 10 to 24 years with newly-diagnosed T2DM were investigated for findings of general inflammation. Anthropometric measurements were taken. Data related to arterial stiffness [brachial artery distensibility (Branch D), augmentation index (AIx), carotid-femoral pulse wave velocity (CF-PWV)] were collected. The subjects were divided into a non-LI group (NLI, n=42) and a LI group (n=56) according to their high-sensitivity C-reactive protein (Hs-CRP) levels.

**Results:**

There were no significant differences in age and gender between the LI group and the NLI group. CF-PWV and AIx values of the LI group were higher than those of the NLI group (p<0.01), while Branch D values were lower in the LI group (p<0.01). Branch D, CF-PWV, and AIx values correlated significantly with Hs-CRP overall (r=-0.32, 0.34, 0.33, all p<0.01). Multivariate models revealed that in either group (LI or NLI), Hs-CRP, as a continuous variable, was an independent determinant of arterial stiffness parameters even after adjusting for other risk factors.

**Conclusion:**

Newly-diagnosed T2DM youth and adolescents with LI present a more adverse cardiovascular disease risk profile and stiffer arteries. Hs-CRP levels correlated with arterial stiffness parameters and constituted an independent determinant of arterial stiffness.

**WHAT IS ALREADY KNOWN ON THIS TOPIC?**

It is well known that adults with type 2 diabetes mellitus (T2DM) are at increased risk to develop cardiovascular disease due to the accelerated atherosclerosis. Adolescents with T2DM also appear to be at higher risk to develop atherosclerosis. Compared to their adolescent counterparts without diabetes, youth and adolescents with T2DM demonstrate increased arterial stiffness, a known precursor of atherosclerosis.

**WHAT THIS STUDY ADDS?**

We investigated whether low-grade inflammation correlates with arterial stiffness in Chinese youth and adolescents with newly-diagnosed T2DM.

## INTRODUCTION

A chronic low-grade inflammation (LI) and an activation of the immune system are observed in abdominal obesity and may have a role in the pathogenesis of obesity-related metabolic disorders, such as metabolic syndrome (MS), insulin resistance (IR), type 2 diabetes mellitus (T2DM), and cardiovascular disease (CVD) (1). It has been shown that systemic inflammatory markers are risk factors for the development of T2DM and its macrovascular complications (2).

With the economic development which took place in China over the past decades, the prevalence of childhood obesity has increased dramatically, ushering a variety of health problems including T2DM, which previously was not typically seen until much later in life (w). It is well known that adults with T2DM are at increased risk to develop CVD due to the accelerated atherosclerosis (4). Adolescents with T2DM also appear to be at higher risk to develop atherosclerosis. Compared to their adolescent counterparts without diabetes, youth and adolescents with T2DM demonstrate increased arterial stiffness, a known precursor of atherosclerosis (5).

To our knowledge, the correlations between LI and arterial stiffness have not been previously studied in Chinese youth and adolescents with newly-diagnosed T2DM. To address this issue, we evaluated arterial stiffness using pulse wave velocity (PWV), brachial artery distensibility (Brach D), and augmentation index (AIx) in a group of Chinese adolescents and young adults with newly-diagnosed T2DM and sought to determine whether high-sensitivity C-reactive protein (Hs-CRP), a marker of LI, correlated with arterial stiffness.

## METHODS

Ninety-eight participants (52 males and 46 females; mean age 18.4±4.2 years) were recruited from among the inpatient and outpatient populations of the departments of Endocrinology and Pediatrics of the Zhongnan Hospital at Wuhan University. Eligibility criteria included presence of a newly-diagnosed T2DM state in subjects aged from 10 to 24 years who were free of the acute complications of diabetes such as diabetic ketoacidosis (DKA), and who also had no history or evidence of infectious diseases, autoimmune diseases, use of glucocorticoids in the past 3 months, and other relevant medical histories. Pregnant females were also excluded from this study. The patients were divided into LI group and non-LI group (NLI), according to their Hs-CRP levels. A cut-off value of 2 mg/L for the Hs-CRP values was accepted to define subjects with LI (6).

The diagnosis of T2DM was based on the American Diabetes Association criteria (7) and the guidelines recommended by the American Academy of Pediatrics (8). Diabetes was defined as: 1) Hemoglobin A1c (HbA1c) ≥6.5% (test performed in an appropriately certified laboratory); or 2) (defined as no caloric intake for at least 8 hours) fasting plasma glucose (FPG) ≥7.0 mmol/L; or 3) 2-hour plasma glucose ≥11.1 mmol/L during an oral glucose tolerance test (OGTT) performed as described by the World Health Organization by using a glucose load containing the equivalent of 75 g anhydrous glucose dissolved in water; or 4) a random plasma glucose level of 11.1 mmol/L with symptoms of hyperglycemia. In the absence of unequivocal hyperglycemia, criteria 1-3 were confirmed by repeat testing. None of the patients had evidence of another specific type of diabetes. All subjects were islet cell antibody-negative (glutamic acid decarboxylase, islet cell antigen, insulin auto-antibodies).

All study procedures were approved by the Human Research Ethics Committee of Zhongnan Hospital, Wuhan University. Prior to enrollment in the study, written informed consent was obtained from subjects ≥18 years old and from the parents or guardians for subjects <18 years old.

All the participants were given a questionnaire which included questions on the name, age, gender, occupation, smoking and drinking history, medical history, and family history of the individual. The average of two consecutive measurements of height and weight were used in the analyses. Body mass index (BMI) was calculated as weight (kg)/height (m)2. The average of 3 measurements of systolic blood pressure (SBP) and diastolic blood pressure (DBP), performed by a registered nurse using a mercurial sphygmomanometer with the subjects in a resting state, was used.

After a minimum 10-h fast, blood samples were collected. FPG was measured by using an Olympus AU5400 automatic biochemical analyzer with intra-assay and inter-assay coefficients of variation of 1.2% and 1.6%. Fasting plasma insulin (FINS) was analyzed by using a Siemens immune 1000 automatic chemiluminescence analyzer. Homeostasis model assessment of IR (HOMA-IR) was calculated as FINS×FPG/22.5. Hs-CRP was measured by using a high-sensitivity enzyme-linked immunosorbent assay. Lipid profiles were performed by using an Olympus AU5400 automatic biochemical analyzer.

### Arterial Stiffness Measurements

Brach D, carotid-femoral PWV (CF-PWV), and AIx were used as measures of arterial stiffness (9). Data of Brach D were obtained by a DynaPulse Pathway Instrument (PulseMetric, San Diego, CA, USA), which derived brachial artery pressure curves from arterial pressure signals obtained from a standard cuff sphygmomanometer assuming a straight tube brachial artery and T-tube aortic system. The coefficients of variability in this study were <9%.

A SphygmoCor SCOR-PVx System (Atcor Medical, Sydney, Australia) was used for heart rate (HR), CF-PWV, and AIx. The distance from carotid to femoral artery was measured to the nearest 0.1 cm twice, was averaged, and entered into the software. A tonometer was used to collect carotid and femoral artery waveforms gated by the R-wave on a simultaneously recorded electrocardiogram. CF-PWV was then calculated as the distance measured above divided by the time delay measured between the feet of the two waveforms reported in m/sec. CF-PWV of each subject was measured three times and averaged. The coefficients of variability in this study were <8%.

AIx is derived from the central pressure waveform by calculating the difference between the main outgoing wave and the reflected wave of the central arterial waveform, expressed as a percentage of the central pulse pressure. AIx was adjusted to a standard HR of 75 beats per minute to eliminate the influence of HR. The intra-class correlation coefficients were 0.7 to 0.9.

### Statistical Analysis

The analyses were performed with SPSS 19.0. The data were expressed as means ± standard deviation (SD) for continuous variables, and as ratios for categorical variables. A t-test for continuous variables and X2 analyses for categorical variables were performed to determine whether differences in mean levels of variables existed between the two groups. Bivariate correlations were calculated between arterial stiffness variables and other covariates overall. General Linear Models were constructed using important covariates from correlation analyses to elucidate if LI was an independent determinate of arterial stiffness. A p value <0.05 was deemed significant statistically.

## RESULTS

### Cardiovascular Disease Risk Factors and Arterial Stiffness Parameters by Groups

There were no significant differences in age and gender between the LI and NLI groups. Compared to the NLI group, CVD risk factors such as BMI, SBP, DBP, HOMA-IR, triglyceride (TG), total cholesterol (TC), and low-density lipoprotein cholesterol (LDL-C) were all increased significantly in the LI group (p<0.05 or p<0.01). The high-density lipoprotein cholesterol (HDL-C) values were alse significantly lower in the of LI group (p<0.01). The differences in FPG and HbA1c between the two groups were not significant. CF-PWV and AIx of LI group were higher than those of the NLI group (p<0.01), while Branch D of the LI group was lower than that of the NLI group (p<0.01), indicating that the arterial stiffness of LI group was higher than that of the NLI group (Table 1).

### Correlations Between High-Sensitivity C-Reactive Protein and Arterial Stiffness Parameters

The three arterial stiffness parameters (Branch D, CF-PWV, and AIx) correlated with each other strongly (for all, p<0.01). Branch D, CF-PWV, and AIx also correlated significantly with Hs-CRP overall (r=-0.32, 0.34, and 0.33; for all, p<0.01). Arterial stiffness parameters also significantly correlated with BMI, SBP, DBP, TG, TC, LDL-C, HDL-C, HbA1c, and HOMA-IR (for all, p<0.05 or p<0.01), after the control of which, the coefficient correlation of Hs-CRP with Branch D, CF-PWV, and AIx was found to be -0.11, 0.13, and 0.13, respectively (for all, p<0.05).

### Multivariable Models for Independent Determinants of Arterial Stiffness

Multivariate models revealed that in either group (LI or NLI), Hs-CRP as a continuous variable was an independent determinant of arterial stiffness, demonstrated as Branch D, CF-PWV, and AIx, even after adjusting for BMI, SBP, DBP, TG, TC, LDL-C, HDL-C, FPG, HbA1c, and HOMA-IR. The other independent predictors of the arterial stiffness parameters are shown in Table 2.

## DISCUSSION

This study demonstrated that a more adverse CVD risk profile and stiffer arteries are found in newly-diagnosed T2DM youth and adolescents with LI. Hs-CRP, the marker of LI, may be especially helpful in predicting increased arterial stiffness in young individuals with newly-diagnosed T2DM. These data also suggest that newly-diagnosed T2DM youth and adolescents require aggressive interventions to prevent atherosclerosis-related diseases. To our knowledge, this is the first report about the relationship between LI and arterial stiffness in young and adolescent T2DM patients.

In adults, the correlation between inflammation and T2DM has been well demonstrated. Subclinical chronic inflammation seems to be an independent risk factor for the development of T2DM. It has been reported that high levels of many inflammatory factors at baseline in diverse human populations are correlated with the incidence of T2DM (10). Prospective studies have identified white blood cell count (11), pro-inflammatory cytokines (12), chemokines (13), and other several indirect markers of inflammation, such as fibrinogen, sialic acid, and plasminogen activator inhibitor-1 (14), as predictors of T2DM. Amongst all inflammatory biomarkers, Hs-CRP measurement stands out as the least expensive, better standardized, and widely available (15). We have therefore, in this study, used Hs-CRP to reflect the status of inflammation in young individuals with newly-diagnosed T2DM and found that the cut-off point of LI was 2 mg/L, a finding in accordance with other reports (6).

It is well known that increased arterial stiffness is an independent predictor of CVD and that it is not related to blood pressure (16). The importance of inflammation on the pathogenesis of arterial stiffness has been widely studied in adults (17,18). Arterial stiffness is associated with increased activity of angiotensin II (Ang II), which results in increased NADPH oxidase activity, reduced NO bioavailability, and increased production of reactive oxygen species (19). Ang II not only activates matrix metalloproteinases which degrade transforming growth factor β (TGF-β) precursors to produce active TGF-β, resulting in increased arterial fibrosis, but also activates cytokines, including monocyte chemoattractant protein-1, tumor necrosis factor-α, interleukin-1 (IL-1), IL-17 and IL-6 (20). There are also some clinical evidence that indicate the association of inflammation with increased arterial stiffness. In a 20-year follow-up from the Caerphilly prospective study, conducted on a predominantly Caucasian cohort of 825 men who underwent baseline and follow-up PWV measurements, it has been demonstrated that the only independent predictors of the PWV at follow-up were pulse pressure, CRP, glucose levels, and waist circumference. Among the clinical variables, cumulative exposure to CRP was the variable with the strongest association (21). We also found in this study that Hs-CRP significantly correlated with arterial stiffness parameters and was an independent determinant of arterial stiffness.

Patients with diabetes carry a two to fourfold increased risk of developing CVD. Patients with T2DM also have other CVD risk factors, such as hypertension, dyslipidemia, obesity, and endothelial dysfunction (22). Not only is the incidence of CVD higher in diabetes, the mortality of the diabetic patients after a cardiac event is also significantly increased in comparison with non-diabetics (23). In the past decades, the incidence of T2DM in children and adolescents has shown a progressive increase (8). It has been demonstrated that children and adolescents with T2DM are at greater risk for the development of atherosclerotic CVD than the general population. Naylor et al (24) reported that carotid intima-medial thickness in adolescents with T2DM was increased as compared to both obese and lean control subjects, while flow-mediated dilation in the T2DM group was significantly decreased compared to that of lean group, suggesting impairment of vascular health in adolescents with T2DM. Urbina et al (25) found that arterial stiffness of adolescents and young adults with obesity-related T2DM was increased and also reported that these abnormalities persisted after controlling for factors such as blood pressure and lipids.

Although some research on the relationship of T2DM and arterial stiffness in adolescents has been conducted, the factors influential in this relationship are largely unknown. In a study on healthy adolescents and young adults, it was reported that although IR is associated with increased arterial stiffness, traditional cardiovascular risk factors, especially obesity and blood pressure, were the major determinants of arterial stiffness in such individuals (26). These authors also found that TG/HDL-C, an estimate of small-dense LDL-C, was an independent determinant of arterial stiffness in adolescents and young adults, especially in obese subjects (27). In young individuals and adolescents with newly-diagnosed T2DM, we are the first to report that LI was an independent determinant of arterial stiffness.

It has been well established that obesity is an independent determinant of CVD. Arterial stiffness has been reported in individuals with obesity or MS, and weight reduction may decrease arterial stiffness (28). In a study aiming to investigate the influence of adiposis on the progression of arteriosclerosis in the early teens, it was found that the changes in BMI values correlated positively with systolic and DBP and with PWV, indicating the tight relationship between obesity and arterial stiffness even in young individuals and in adolescents (29). It should be mentioned that BMI and Hs-CRP were both independent predictors of the arterial stiffness parameters in multivariate models in this present study (as shown in Table 2). We also found that the BMI correlated significantly with Hs-CRP levels (data not shown). However, after controlling for BMI and other risk factors, Hs-CRP still correlated with Branch D, CF-PWV, and AIx significantly, and in multivariate models, Hs-CRP was an independent determinant of Branch D, CF-PWV, and AIx even after adjusting for BMI and other factors. These results indicated that Hs-CRP correlated with arterial stiffness parameters and was an independent determinant of arterial stiffness. To measure Hs-CRP may be a useful tool to screen for those at higher risk for CVD among the newly-diagnosed T2DM youth and adolescents.

We realize that this study has some limitations. The smallness of the sample size is one of these limitations. Our cross-sectional design does not allow us to determine whether Hs-CRP is a causal factor of arterial stiffness or just a satellite phenomenon. In addition, we did not assess other “non-traditional” risk factors which may also contribute to increased vascular stiffness such as small LDL particles, lipoprotein (a), serum homocysteine, and endothelin-1.

On the whole, the findings of this study have revealed that newly-diagnosed T2DM youth and adolescents with LI had a more adverse CVD risk profile and stiffer arteries. Hs-CRP correlated with all three arterial stiffness parameters, namely, Branch D, CF-PWV, and AIx, and was an independent determinant of arterial stiffness after adjustment for BMI, SBP, DBP, TG, TC, LDL-C, HDL-C, FPG, HbA1c, and HOMA-IR. The findings also showed that Hs-CRP, the marker of LI, may be helpful in predicting increased arterial stiffness in newly-diagnosed T2DM youth and adolescents.

## Figures and Tables

**Table 1:**
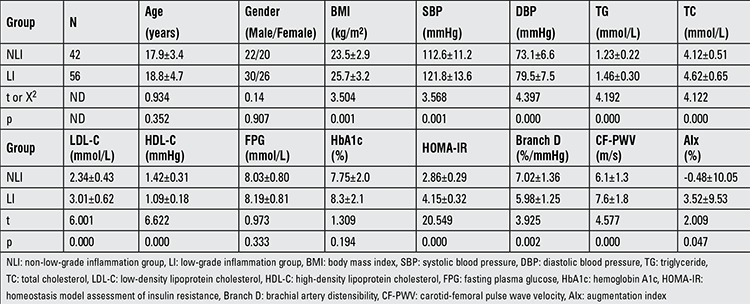
Cardiovascular disease risk factors and arterial stiffness parameters by group (mean ± standard deviation

**Table 2:**
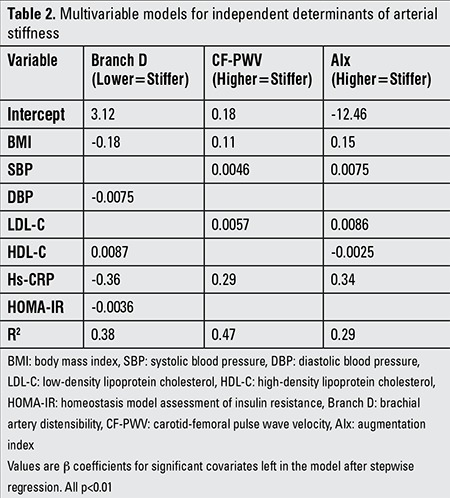
Multivariable models for independent determinants of arterial stiffness
